# Lung ultrasonography as a tool to guide perioperative atelectasis treatment bundle in head and neck cancer patients undergoing free flap reconstructive surgeries: a preliminary observational study

**DOI:** 10.1016/j.bjorl.2020.05.030

**Published:** 2020-07-29

**Authors:** Nitika Goel, Indu Mohini Sen, Jaimanti Bakshi

**Affiliations:** a Postgraduate Institute of Medical Education and Research, Department of Anaesthesia and Intensive Care, Chandigarh, India; b Postgraduate Institute of Medical Education and Research, Department of Otolaryngology, Chandigarh, India

**Keywords:** Lung ultrasound, Atelectasis, Head and neck cancer surgery

## Abstract

**Introduction:**

General anesthesia causes pulmonary atelectasis within few minutes of induction. This can have significant impact on postoperative outcome of cancer patients undergoing prolonged reconstructive surgeries.

**Objective:**

The purpose of this study was to evaluate the impact of sonographically detected perioperative atelectasis on the need for postoperative oxygen supplementation, bronchodilator therapy and assisted chest physiotherapy in patients undergoing free flap surgeries for head and neck carcinoma.

**Methods:**

Twenty eight head and neck cancer patients underwent bilateral pulmonary ultrasonographic assessments before and after lung surgery. Lung ultrasound scores, serum lactate, and PaO_2_/FiO_2_ ratio were measured both at the beginning and at end of the surgery. Patients were scanned in the supine position and the number of single and confluent B lines was noted. These values were correlated with the need for oxygen therapy, requirement of bronchodilators and total weaning time to predict the postoperative outcome. Other factors affecting weaning were also studied.

**Results:**

Among twenty eight patients, seven had mean lung ultrasound score of ≥10.5 which correlated with prolonged weaning time (144.56 ± 33.5 min vs. 66.7 ± 15.7 min; *p* = 0.005). The change in lung ultrasound score significantly correlated with change in PaO_2_/FiO_2_ ratio (*r* = −0.56, *p* = 0.03). Elevated total leukocyte count >8200 μL and serum lactate >2.1 mmoL/L also predicted prolonged postoperative mechanical ventilation.

**Conclusion:**

This preliminary study detected significant levels of perioperative atelectasis using point of care lung ultrasonography in head and neck cancer patients undergoing long duration surgical reconstructions. Higher lung ultrasound scores highlighted the need for frequent bronchodilator nebulizations as well as assisted chest physiotherapy and were associated with delayed weaning. We propose more frequent point of care lung ultrasonographic evaluations and use of recruitment maneuvers to reduce the impact of perioperative pulmonary atelectasis.

## Introduction

Atelectasis is an under-reported perioperative pulmonary complication after positive pressure ventilation. The incidence varies from 50% to 90% in adults undergoing general anesthesia, when spontaneous or positive pressure mechanical ventilation is used.^1^, ^2^ Head and neck cancer reconstruction surgeries are time- consuming, involving adults who are chronic smokers with multiple comorbidities. Accordingly anesthesia is prolonged and patients are put on elective mechanical ventilation in the postoperative period.^3^ The need of positive pressure mechanical ventilation invariably results in collapse of small airways and/or the whole acinus. This loss of lung aeration can vary from local hypoventilation to complete atelectasis,^4^ and is expected to effect the process of weaning from mechanical ventilation. The use of lung ultrasound for detecting anesthesia-induced atelectasis has arisen as a reliable, point of care and non-invasive tool for anesthesiologists.^4^ However, the role of lung ultrasonography in detecting atelectasis in free flap reconstructive surgeries has not been studied. The primary outcome was to study the variation in lung ultrasound scores and to measure the weaning time from mechanical ventilation and requirement for postoperative atelectasis treatment bundle in head and neck cancer patients undergoing reconstructive surgeries. It was hypothesized that lung ultrasonography would be feasible in all patients in detecting perioperative pulmonary atelectasis and subsequently predicting the postoperative outcome in the form of weaning from mechanical ventilation and requirement of bronchodilator therapy and chest physiotherapy.

## Methods

This was a single-center, prospective observational study to evaluate the role of lung ultrasonography in detecting perioperative atelectasis and subsequently predict the postoperative course of mechanical ventilation in head and neck cancer reconstructive surgeries.

The study was conducted in accordance to the principles of Declaration of Helsinki. After obtaining institute ethical committee approval, study was registered with Central Trial Registry India (CTRI/2017/06/008949).

Adult patients aged 18–65 years of either gender, scheduled for elective head and neck cancer resection surgery followed by free flap reconstruction, were included in the study. Patients were thoroughly evaluated one day prior to surgery and written informed consent was taken. Need for admission to high dependency units and postoperative ventilatory support was explained. All patients were premedicated with oral alprazolam 0.25 mg and ranitidine 150 mg the night before surgery and again 60–90 min before the expected time of surgery. In the operating room, an intravenous line was secured and injection of glycopyrrolate 0.2 mg was administered intravenously to all patients. Baseline monitoring consisted of electrocardiography, non-invasive blood pressure monitoring, capnography, pulse oximetry, temperature and urine output monitoring.

In all patients, oxygen supplementation was given and definitive airway in the form of surgical tracheostomy was created under local anesthesia. Thereafter, general anesthesia was induced using morphine 0.1 mg/kg, propofol 2.0 mg/kg and vecuronium 0.1 mg/kg to facilitate muscle relaxation. Mechanical ventilation was standardized for all patients. GE Datex-Ohmeda Aestiva 3000 (GE Healthcare, Wauwatosa, WI) delivered volume-controlled ventilation (tidal volume 8 mL/kg of predicted body weight, FiO_2_ 0.40, respiratory frequency 12 breaths/min adjusted to obtain an end-tidal carbon dioxide between 32 and 36 mmHg, inspiratory to expiratory ratio of 1:2, and positive end-expiratory pressure (PEEP) 5 cmH_2_O. Anesthesia was maintained using isoflurane 1–1.2% minimum alveolar concentration with 30% oxygen and 70% nitrous oxide mixture. Supplemental doses of vecuronium (one-fourth of the initial bolus dose) were administered as per requirement. Radial artery cannulation was done for continuous invasive blood pressure monitoring and for taking samples for blood gas monitoring. A wide bore 16G intravenous line preferably was also secured to manage massive fluid shifts.

Data collected included demographic characteristics, comorbidities, routine blood parameters, and intraoperative hemodynamics including heart rate, blood pressure, capnography, pulse oximetry, minimum alveolar concentration and temperature monitoring throughout the length of surgery. The patient's hemoglobin, serum lactate and PaO_2_/FiO_2_ levels were measured both at the beginning and at end of the surgery. The total duration of surgery, perioperative fluid administration, urine output and estimated blood loss was also recorded. The requirement of any blood transfusion and vasopressor support and use of diuretics intraoperatively was also recorded.

**Figure 1:**
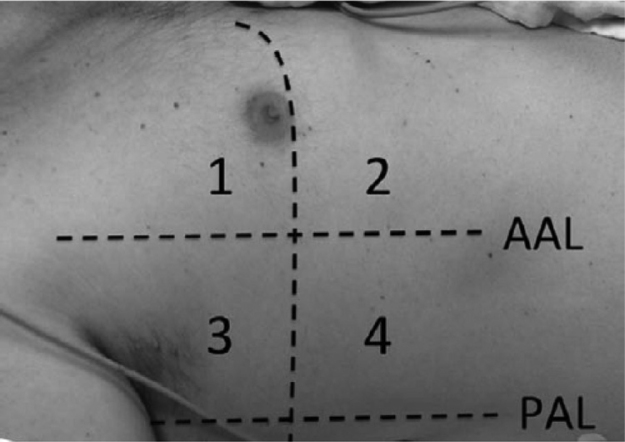
Each hemithorax is divided into four quadrants by anterior axillary line (AAL, anterior axillary line; PAL, posterior axillary line).

Lung ultrasound was performed by two trained anesthesiologists (with minimum of six months experience in lung ultrasonography). The Sonosite ultrasound machine with a 3–5 MHz curved array probe (GE Healthcare) was used for all lung USG examinations. Lung aeration scores were based on a previous study by Montesse et al., in which the thorax was divided into six quadrants on each side – anterior, lateral and posterior by anterior and posterior axillary lines.^5^ However since our patients had free flap reconstruction in the postoperative period, the feasibility of scanning the posterior quadrants was not possible. Therefore we calculated the lung ultrasound score by dividing the thorax into only four quadrants on each side – anterior and lateral by anterior axillary line (Fig. 1). Patients were scanned while in the supine position and two sets were recorded for all the patients, one before surgery and one at the completion of surgery. Intercostal spaces (ICS) of each of these areas were scanned. The number of single and confluent B lines was recorded, and a score ranging from 0 (no aeration loss) to 24 (complete aeration loss) was calculated to summarize the B lines of the eight ICSs (Table 1). All examinations were performed by an independent observer unaware of the previous ultrasound findings within time interval of 5–8 min.

**Table 1:** Original lung ultrasound scoring system.

	Normal aeration	Mild loss of aeration	Moderate loss of aeration	Severe loss of aeration
Quotation	0	1	2	3
Original lung ultrasound score	0–2 B lines	≥3 B lines	Multiple coalescent B lines	Consolidation

The number of single and confluent B lines was recorded, and a score ranging from 0 (no aeration loss) to 24 (complete aeration loss) was calculated to summarize the B lines of the eight ICSs.

Just before skin closure, a gastric feeding tube was inserted in all patients. Patients were then transported to the high dependency unit in a sedated state where they were mechanically ventilated via the newly-created tracheostomy. On the morning of postoperative day 1, sedation was withdrawn and patients were allowed to emerge slowly from anesthesia. They were transitioned to pressure support ventilation or continuous positive airway pressure mode and finally weaned from mechanical ventilation. Total weaning time (time patient remains on weaning mode) was subsequently recorded. Once the patient started maintaining spontaneous breathing on T-piece, decision to shift to the ward was taken. In the ward, total duration of oxygen requirement through T-piece for maintaining SpO_2_ > 92%, along with need for bronchodilator and chest physiotherapy was recorded.

The sample size calculation was based on the previous study,^5^ the association between differences in LUS scores (preoperative versus postoperative) and change in partial pressure of arterial oxygen PaO_2_/FiO_2_ ratio. Assuming that the correlation between the two factors was 0.45 and power of 0.8, a sample size for the group was determined to be 29, as calculated by correlation. The sample size for enrollment was 29. The normality of the measurable data was assessed using the Kolmogorov–Smirnov test. The normally distributed data was expressed as mean, standard deviation; range etc. whereas skewed data was expressed as median and inter quartile range. For time-related comparisons, student's *t*-test (paired) or Wilcoxon signed rank test was applied for normal or skewed data respectively. The categorical data was expressed as frequencies, percentages etc. The association of various categorical/classified data was analyzed using Chi-square test or Fisher's exact test, whichever was applicable. Pearson correlation was used to assess association between the oxygenation ratio and lung ultrasound scores. The correlation between various patient parameters and lung ultrasound scores was also studied. Logistic regression analysis was done to assess various factors predicting delay in weaning from mechanical ventilation and subsequent oxygen requirement. A *p*-value of <0.05 was considered significant in all the tests. Analysis was performed using Excel 2007 (Microsoft, Redmond, WA, USA) and SPSS version 15.0 software (IBM, Armonk, NY, USA).

## Results

**Figure 2:**
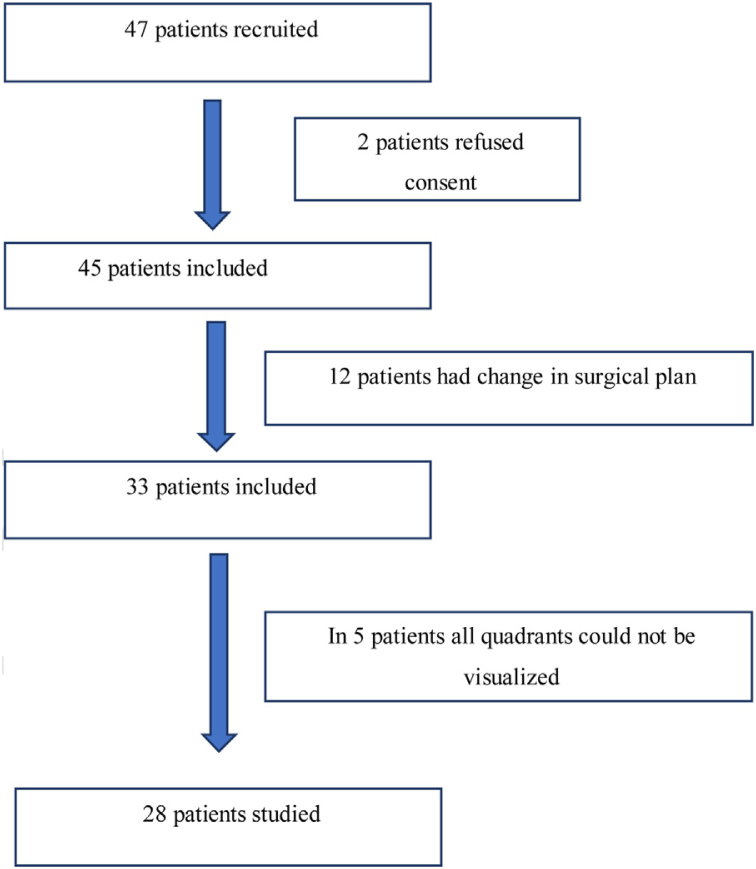
Consort diagram.

Forty-seven adult patients met the eligibility criteria in the six months enrollment period (May 2017 to November 2017). Two patients refused to participate in the study. In twelve patients the surgical plan was changed from free flap reconstructive surgery to primary closure. In another five patients, all lung zones could not be visualized. The consort diagram has been shown in Fig. 2. The demographic profile of twenty eight patients who completed the study is shown in Table 2.

**Table 2:** Demographic features of study population.

*Total*	28 (100)
*Male (%)*	26 (92.9)
*Female (%)*	2 (7.1)
*Weight, (mean* *±* *SD), kg*	66.5 ± 9.5
*Smoking history+(%)*	18 (64.3)
*Alcoholic history+(%)*	14 (50)

*Diagnosis*	
Ca alveolus (%)	15 (53.5)
Ca buccal mucosa (%)	11 (39.2)
Ca oropharynx (%)	1 (3.5)
Low grade mucoepidermoid carcinoma of SMG (%)	1 (3.5)

*Comorbidities, no (%)*	
Hypertension (%)	12 (42.8)
DM (%)	6 (21.4)
COPD (%)	2 (7.1)

*Prior radiotherapy (Yes/No) (%)*	13 (46.4)/15 (33.6)
*Prior tracheostomy (Yes/No) (%)*	8 (28.6)/20 (71.4)
*Stage of tumor (IV/III/II) (%)*	18 (64.3)/8 (28.6)/2 (7.1)

*Reconstruction pectoralis flap (%)*	
Radial bone free flap (%)	22 (80.9)
Rectus abdominis flap (%)	4 (16)
Fibular free flap (%)	1 (1.8)
Radial bone free flap (%)	1 (1.8)

Values are expressed as absolute numbers (%).

The change in lung ultrasound score significantly correlated with change in PaO_2_/FiO_2_ ratio (*r* = −0.56, *p* = 0.03), recorded at beginning and end of surgery. The duration of surgery also correlated with changes in lung ultrasound scores (*r* = −0.66, *p* = −0.001).

The presence of lung ultrasound score >10.5 ± 0.17 at the end of surgery predicted prolonged weaning time and requirement of postoperative bronchodilator therapy, chest physiotherapy and additional oxygen supplementation with both sensitivity and specificity of 100%. The area under receiver operator characteristic curve was 1.0. Among the twenty-eight patients, seven patients had a mean lung ultrasound score of ≥10.5, which correlated with prolonged weaning time (Pearson correlation coefficient *r* = 0.68, *p* = 0.007). The average weaning time in these seven patients was 144.56 ± 33.5 min vs. 66.7 ± 15.7 min in patients with lower ultrasound scores (*p* = 0.005). One patient out of these seven also required vasopressor support, from which was weaned after 48 h of shifting to recovery. The mean duration of total ventilator stay was 900.45 ± 101.2 min vs. 840.34 ± 113.54 min with lower lung ultrasound scores (*p* = 0.234). There was no significant difference in total ventilator stay in patients with lower or higher lung ultrasound scores. All the seven patients were shifted to the surgical ward on the second postoperative day and required oxygen supplementation for 3–5 days. The patients also required additional nebulizations with salbutamol and assisted chest physiotherapy. The average postoperative stay in hospital in patients with higher lung ultrasound scores was 21 ± 3.5 days as compared to those with less ultrasound scores (11 ± 2.4 days, *p* = −0.008). The postoperative 30-day survival rate was 100% in patients with and without prolonged mechanical ventilation. The comparison of demographic profile, comorbidities, surgical factors and postoperative outcome in patients with lower and higher lung ultrasound scores is shown in Table 3, Table 4, Table 5.

**Table 3:** Comparison of demographic characteristics in patients with high and low lung ultrasound scores.

	Lung ultrasound score (<10.5)	Lung ultrasound score (>10.5)	*p*-Value
	*n* = 21	*n* = 7	
Age (yrs)	52.6 ± 10.3	54.6 ± 11.3	0.816
BMI (kg/m^2^)	24.33 ± 11.3	25.39 ± 10.3	0.282
Sex (M/F)	20/1	6/1	0.113
ASA status (III/II/I)	1/17/3	3/3/1	0.231
Stage of tumor (IV/III/II)	13/6/2	4/2/1	0.322
Comorbidities	5	2	0.231
Smoker (Yes/No)	13/7	5/2	0.294
Alcohol (Yes/No)	11/10	3/4	0.454
Prior radiotherapy (Yes/No)	9/12	4/3	0.456
Prior tracheostomy (Yes/No)	4/17	3/4	0.235
Hb (baseline) (g/dL)	12.3 ± 2.4	11.8 ± 1.9	0.678
TLC	7800 ± 1100	9200 ± 1029	0.115
Platelet count (lacs/L)	1.23 ± 0.98	1.16 ± 0.72	0.222
Blood urea (mg/dL)	18 ± 2.3	20 ± 1.8	0.345
S creatinine (mg/dL)	1.12 ± 0.6	1.03 ± 0.8	0.245
S electrolytes (mEq/dL)	134 ± 5.6/4.5 ± 0.78	136 ± 4.8/4.4 ± 0.45	0.435
S bilirubin (mg/dL)	0.34 ± 0.11	0.24 ± 0.06	0.675
SGOT (IU/L)	45 ± 10.9	44 ± 11.2	0.523
SGPT (IU/L)	35 ± 12	40 ± 11	0.365
S albumin (g/dL)	3 ± 0.45	3.2 ± 0.67	0.456
Blood sugars (mg/dL)	145 ± 5.6	135 ± 5.8	0.876

Smoker included current smokers or those with ≥10 pack years of smoking, alcoholic included were those with history of consumption from past more than 10 years.

**Table 4:** Comparison of surgical factors in patients with high and low lung ultrasound scores.

	Lung ultrasound score (<10.5)	Lung ultrasound score (>10.5)	*p*-Value
	*n* = 21	*n* = 7	
Duration of surgery (min)	433.57 ± 101.3	728.57 ± 119.8	0.001
Total crystalloids (mL)	3557.14 ± 1000.7	3856.23 ± 879.7	0.358
Total colloids (mL)	333.67 ± 110.1	400.23 ± 123.5	0.236
PRBC/WBC/FFP/platelet transfusion (mL)	350.25 ± 98.23	389.6 ± 101.2	0.234
Urine output (mL)	647.32 ± 108.78	768.45 ± 110.34	0.173
Lasix (mg)	8.33 ± 2.35	9.45 ± 1.34	0.234
Estimated blood loss (mL)	759.89 ± 110.34	850.45 ± 109.45	0.289
Hb (end) (g/dL)	10.29 ± 2.34	9.23 ± 1.45	0.17
Lactate (pre) (mmoL/L)	1.24 ± 0.56	1.13 ± 0.67	0.565
Lactate (end) (mmoL/L)	2.08 ± 1.34	2.56 ± 1.45	0.476
PaO_2_/FiO_2_ (Pre)	427.27 ± 101.23	445 ± 122.23	0.426
PaO_2_/FiO_2_ (end)	337.24 ± 109.67	137.29 ± 56.8	0.000

**Table 5:** Comparison of postoperative parameters in patients with high and low lung ultrasound scores.

	Lung ultrasound score (<10.5)	Lung ultrasound score (>10.5)	*p*-Value
	*n* = 21	*n* = 7	
Duration of postoperative mechanical ventilation (min)	840.34 ± 113.54	900.45 ± 101.2	0.234
Total weaning time (min)	66.7 ± 15.7	144.56 ± 33.5	0.005
Duration of postoperative oxygen therapy (days)	–	4.25 ± 1.2	0.002
Duration of hospital stay (days)	10.5 ± 2.4	22.5 ± 4.6	0.008

**Figure 3:**
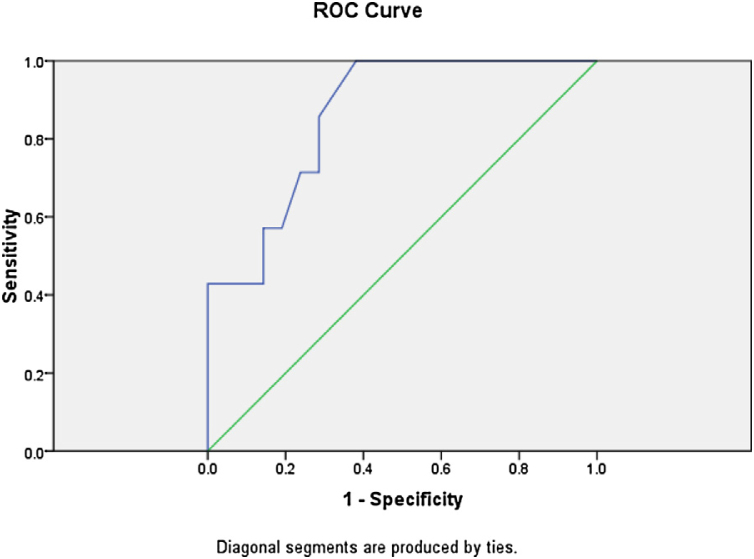
The receiver operator characteristic curve for total leukocyte count is shown with area under curve of 0.861.

**Figure 4:**
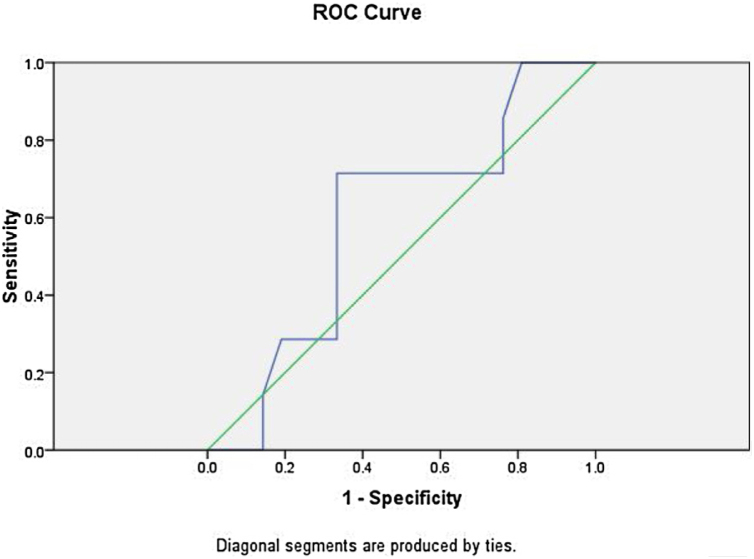
The ROC curve for serum lactate is shown with area under curve of 0.592.

Logistic regression analysis was done to analyze various factors involved in postoperative weaning from mechanical ventilation and subsequent oxygen supplementation. On multivariate regression analysis an elevated total leukocyte count >8200 μL and serum lactate >2.1 mmoL/L predicted the requirement of postoperative mechanical ventilation and further oxygen supplementation with sensitivity of 85.7% and specificity of 90%. The receiver operator characteristic curve for total leukocyte count showed area under curve of 0.861 (Fig. 3). The ROC curve for serum lactate showed area under curve of 0.592 (Fig. 4).

## Discussion

General anesthesia causes atelectasis within the first few minutes of induction in most dependent parts of lung.^6^, ^7^ In the supine position, approximately, 15–20% of lung tissue near the diaphragm or about 10% of total lung mass may develop atelectasis during surgery, which may extend up to 50% of lung tissue in open heart surgery.^8^ Mild to moderate perioperative atelectasis usually occurs in nearly half of elective surgical patients, approximately 20% patients may develop severe atelectasis (oxygen saturation < 81% up to 5 min) intraoperatively^9^ and 13% in the post-anesthesia care unit.^10^ Perioperative atelectasis, though a self-limiting lung aberration, can progress to a critical level in high-risk patients. The initial presentation is hypoxemia, which can progress to pneumonia as a continuum of intraoperative atelectasis.^11^

In this preliminary observational study, the prevalence of perioperative pulmonary atelectasis detected by lung ultrasonography was 25%. None of the patients progressed to pneumonia in the postoperative period. However, the patients who developed atelectasis during the course of surgery not only had prolonged weaning from ventilator but also required bronchodilator therapy, chest physiotherapy and oxygen support through a T-piece to maintain SpO_2_ > 92% in the postoperative period for next 3–5 days.

The medical literature documents various studies where lung ultrasonography has been used as a useful tool for detecting perioperative atelectasis with sensitivity and specificity of 93% and 100%, respectively.^12^ Acosta et al. in their study have demonstrated lung ultrasound as an accurate, safe, and simple bedside method for diagnosing atelectasis in anesthetized children.^13^ In their study, using magnetic resonance imaging as the standard, bedside ultrasound had a sensitivity of 88% and specificity of 89% for the diagnosis of anesthesia-induced atelectasis. In a study by Yu et al. they postulate that that LUS scores highly correlate with the atelectasis volume of computed tomography (*r* = 5–0.58, *p* = −0.0001).^14^ Yu et al. demonstrated that using computed tomography as standard, with a sensitivity of 87.7%, specificity of 92.1% and diagnostic accuracy of 90.8%.

Monastesse et al. demonstrated moderate correlation in the LUS score between the postinduction period and arrival in the recovery room with changes in oxygenation (Spearman *r* = −0.43, *p* = 0.018). Induction of GA was associated with an increase in the LUS score, which gradually worsened at all time points until recovery room discharge. This increase was significantly worse in the basal and dependent lung zones. In their study lung ultrasonography helped in the detection of 2 capnothoraces, 1 endobronchial intubation, and 1 episode of subclinical pulmonary edema.^5^

Since head and neck cancer reconstruction surgeries are prolonged duration surgeries with planned postoperative mechanical ventilation support, these surgeries have a high incidence for perioperative atelectasis. Based on prior literature, the presence of small subpleural consolidations are also one echographic manifestation of atelectasis: small hypodense areas abutting the pleural line similar to “true” consolidations but smaller and devoid of their characteristic tissular pattern,^15^ and displaying at least one B line at its inferior border can develop. Furthermore, increasing PEEP causes the disappearance of these small subpleural consolidations.^16^

During surgery, atelectasis may cause intraoperative gas exchange abnormalities, which may be increased by inflammation triggered by the surgery itself, leading to postoperative lung dysfunction, even in patients without preexisting lung injury. Therefore, we attempted to measure these changes using lung ultrasound and correlate them with the subsequent patient outcome. Though studies are present in which lung ultrasound has been used to assess the diaphragm motion and correlate with pulmonary functions in postoperative period,^17^, ^18^ no previous study has documented the role of serial lung ultrasonography in predicting the postoperative outcome.

Computed tomography (CT) measured lung aeration has been the gold standard for the study of perioperative atelectasis.^4^ However, cumulative radiation exposure^19^ and the need to transport the patient to or from the radiology department limits its use even in the research setting. Ideally suited to study perioperative aeration loss, lung ultrasonography allows imaging the patients at multiple time points in the operating room even during ongoing surgery.^5^

**Figure 5:**
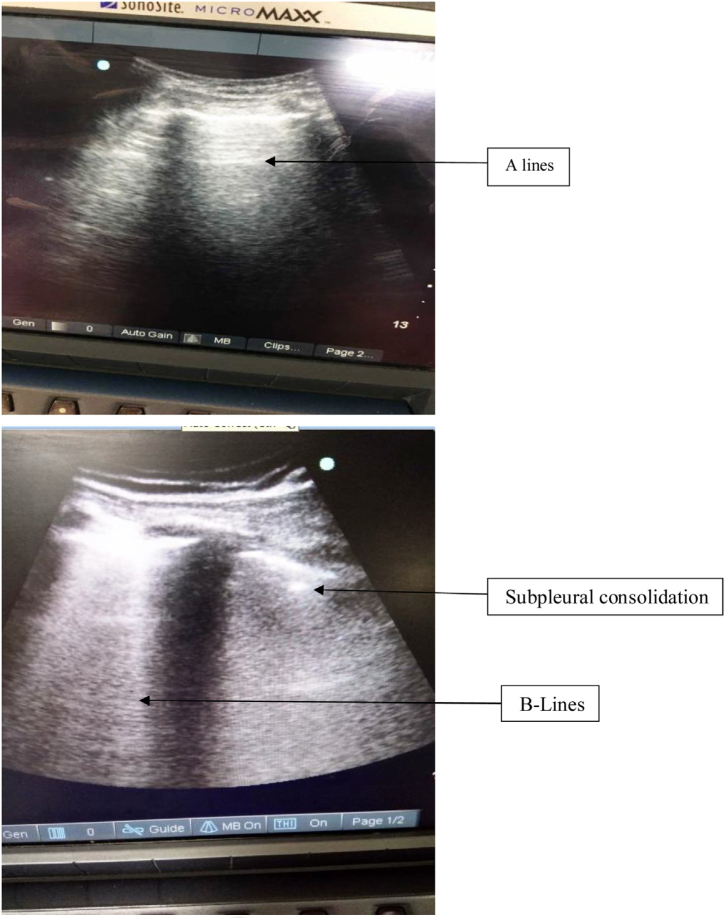
Lung ultrasonography of right upper and lower quadrants. Lower quadrant shows the presence of subpleural consolidation and multiple B lines.

It is to be noted that the presence of B lines have also been used for diagnosis of alveolar interstitial syndrome.^20^ The presence of diffuse comet tail B lines has been hallmark of increased extravascular lung water content.^4^ However the presence of comet tail artifact only in the last intercostals spaces can be seen in normal lungs, their presence diffusely throughout the lungs is helpful in diagnosing pulmonary edema, pulmonary fibrosis and ARDS.^20^ Fig. 5 shows the presence of consolidation in inferior quadrants with normal A lines in the upper quadrant of the same patient in our study. In our study, most of the patients had presence of B lines limited to inferior quadrants only. Also the fluid administration was titrated according to the pulse pressure variation in all patients. Therefore, the chances of increased extravascular lung water content were minimal.

Besides lung ultrasonography, PaO_2_/FiO_2_ levels are also used as surrogate markers for perioperative atelectasis.^21^ The presence of atelectasis (higher lung ultrasound score) also correlated with lower postoperative PaO_2_/FiO_2_ ratio. However, PaO_2_/FiO_2_ ratio alone did not show significant correlation with the weaning time from mechanical ventilation. Esteve et al. in their study has evaluated the usefulness of PaO_2_/FiO_2_ ratio to predict mortality in patients immediately after cardiac surgery.^22^

The presence of smoking status also did not predict the postoperative ventilator requirement and subsequent oxygen requirement in the ward. Preoperative smokers were also found to have the same risk as that of non-smokers in their requirement of postoperative mechanical ventilation. This was in accordance with the study by Petrar et al. Their study demonstrated equal risk of postoperative pulmonary complications in both smokers and non smokers.^23^

Our study also showed elevated total leukocyte count >8200 μL and serum lactate >2.1 mmoL/L has sensitivity and specificity in predicting prolonged weaning time by 85% and 90% respectively. Since total leukocyte count is one of the early markers for sepsis, their elevation could also be due to underlying infection.^24^ Also the elevated serum lactate levels, which are early biomarkers for organ dysfunction, alone can only detect underlying decreased organ perfusion.^25^ However the combination of both parameters could be helpful in detecting the underlying organ disease and postoperative recovery.

This study should be read in light of a few limitations. Firstly, only two reading of lung ultrasonography were planned. We feel that lung ultrasonography could be done more frequently, especially during the intraoperative and postoperative periods until complete recovery takes place. Secondly, we could not visualize the posterior and dorsal parts of the lung, the region where maximum atelectasis occurs. This was due to presence of flap reconstruction on the head and neck area which had to be kept immobile during first 24 h post surgery, thus preventing the patient from being turned laterally for lung ultrasonography. This might have lead to underscored lung ultrasound score values. Thirdly, the lung oxygenation index PaO_2_/FiO_2_ was not recorded in the weaning phase, though they were performed during assessment for weaning. However, this limitation was applicable to all patients. The strength of the study is that the same groups of patients were evaluated and all the surgeries were performed by same team of surgeons with more than 10 years experience.

## Conclusion

This preliminary observational study detected significant perioperative pulmonary atelectasis in patients undergoing free flap reconstructive surgeries for head and neck carcinoma using point of care lung ultrasonography. Furthermore, the presence of higher ultrasound scores after the completion of surgery can be taken as surrogate marker for prolonged weaning, frequent postoperative requirement of bronchodilators chest physiotherapy and oxygen supplementation for a favorable outcome of the patient. Though further trials in larger number of patients are needed, we propose lung ultrasonographic examinations at regular intervals and intermittent lung recruitment manuvers in head and neck cancer patients undergoing long-duration surgeries.

## Conflicts of interest

The authors declare no conflicts of interest.
